# Notes on Some Bristol Medical Societies

**Published:** 1914-12

**Authors:** G. Munro Smith

**Affiliations:** Consulting Surgeon to the Bristol Royal Infirmary


### NOTES ON SOME BRISTOL MEDICAL SOCIETIES.

BY

G. MUNRO SMITH, M.D. Bristol,

*Consulting Surgeon to the Bristol Royal Infirmary.*

DURING my investigations into the history of the Bristol Royal Infirmary I have come across many references to societies and clubs founded in Bristol during the eighteenth and nineteenth centuries. Several of these were Book clubs, formed for the purpose of circulating medical and surgical books amongst the members ; others were for the discussion of pathological and clinical matter, for reading papers on scientific subjects, and so forth. Nearly all were strengthened and made more interesting by a social element, in the form of an occasional dinner or supper at private houses, or at some well-known tavern, etc.

It seemed to me that these notes were worth a permanent record, not only because they throw light on medical activities in our city, but because they give some idea of social life during an interesting period, and add something to our knowledge of many Bristol practitioners whose work should not be forgotten.

I am indebted to that storehouse of information, the *Richard Smith Memoirs*, for the greater part of the facts I have put together; also to old Bristol newspapers and articles scattered through medical and other journals.

### A Bristol Medical Society was founded about the year 1788. The meetings were held in the Committee Room of the Infirmary.

Mr. David Davies, writing to Richard Smith in 1831, says that it "originated in a friendly feeling subsisting at that time between the following members and some others, whose names at present I do not recollect: Brickenden(1), Dyer(2), Dyer(3), Fox(4), Griffiths(5), Lax(6), Maurice(7), Jardine(8), and Davies(9). It was dissolved in 1793 or 1794 by the sale of the books which had been purchased: but the Donations, of which we had several, were placed in the hands of the Treasurer until another Society should be formed. Medical papers were read and discussed, and the members occasionally partook of a supper.—D. D., 1 June, 1831."

(1) Thomas Brickenden, apprenticed to Mr. Thomas Griffiths, and a pupil of Mr. Noble. He had a large practice afterwards in London, and is mentioned by Sir Astley Cooper in his work on Hernia.

(2) Probably W. Dyer, apothecary, uncle of (3) T. W. Dyer, elected Apothecary to the Infirmary, 1759.

(4) E. Long Fox, Physician to Bristol Royal Infirmary, 1786–1816.

(5) Thomas Griffiths, elected Apothecary to the Infirmary, Dec. 2nd, 1783.

(6) Robert Lax, a greatly-esteemed practitioner in Queen's Square. Became Mayor of Wells.

(7) William Maurice, pupil of Mr. Hetling. Became Army surgeon, and received medal for services at Quatre Bras and Waterloo.

(8) Lewis John Jardine. Apprenticed to Mr. Yeatman, 1781. Elected Surgeon to St. Peter's Hospital, Feb. 14th, 1788. Twice applied for Surgeoncy at Infirmary. Died about 1824.

(9) David Davies. Had a large practice in Bristol. Married Miss Dolman of Stokes Croft in 1783. One of his bills for attendance on Mr. Catcott is in existence. The items are chiefly "electuaries" and draughts. There is a note written in Richard Smith's *Memoirs* as follows: "Mr. Nelson, with whom Mr. Davies was, recommended him to stick up to Miss Dolman, who had good property. Mr. Davies said, 'O Sir, I cannot, I have no pretensions.' 'Pretensions,' replied Mr. Nelson, 'nonsense, why you are a good handsome fellow. I will lend you £30 to go a-courting.'"

Probably this was revived, for there is in existence a list headed "Bristol Medical Society," which was found fastened in a book, [1] published in 1805, evidently belonging to some book club. It contains ten names: Messrs. Shute, Lassalle, Rolfe, Wallis, Phillips, Prowse, Rich, Hill, Williams and Allen.

There is another list, belonging to some circulating book club, with the date 1796–97, with twelve names, viz.: Beddoes, Stephen, Crawford, Nott, Renaudet, Fox, Cox, Moncrieffe, Plomer, Lovell, Carrick, and Burrow. As six of these were at different dates physicians to the Infirmary (Crawford, Fox, Moncrieffe, Plomer, Lovell and Carrick), I think it probable that this was the

### Infirmary Medical Reading Society, which was in existence as late as 1819, and consisted then of people connected with the Infirmary (but not at that date on the Hon. Staff), together with other practitioners.

Dr. Renaudet was an American Refugee who lived in Dowry Parade. He led an idle life, doing little but take short walks in sunny weather, "with a muff on his hands and a Spanish lap dog under his arm." Richard Smith, senior, called on him one morning at eleven o'clock and found the doctor still in bed. In a cupboard in the room he saw an object which

[1] In the possession of Dr. A. B. Prowse, November 30th, 1893.

puzzled him, and on inquiry was told that it was a boy airing Renaudet's wig; he did this by wearing the large peruke every day for two hours, in order that it might be properly warmed by a "gradually acquired animal heat."

### The Medical Book Society.

was founded on January 1st, 1796, chiefly by the organisation of Francis Cheyne Bowles, at this time a young man of 25 years of age, full of energy, and a strong advocate of the rational teaching of anatomy by dissection. He was appointed Treasurer of the Club at its first meeting. The original members were: Thomas Baynton, Thomas Griffiths, W. Henry Goldwyer, S. S. Salmon, Thomas Shute, David Davies, Richard Smith, T. W. Dyer, James Dew, John Newman, Samuel Williams, and Francis Cheyne Bowles.

Thomas Baynton was in busy practice in the house which was then "No. 50 in the Old Market, within a few doors of the turning by the Chappel into Jacob Street." He was at this time 34 years of age, and was widely known.

W. Henry Goldwyer became famous as the founder of and first surgeon to the Hospital for Diseases of the Eye in Maudlin Street.

S. S. Salmon was born on February 8th, 1768. He applied twice for the Surgeoncy of the Infirmary.

The first copy of the rules, written on January the 1st, 1796, states: That the objects of the Society were for "promoting a friendly intercourse among its members, and purchasing medical books."

The meetings were to be held at members' houses, in rotation, on the first Friday in every month, at six o'clock "to take tea and converse on medical subjects." There was an annual subscription of half a guinea, and at the end of the year the books were to be sold by auction "to the best bidder"; and "such books as will not produce half the prime cost become the property of the member who propos'd them at that price."

Each book was to be kept a certain number of days, and the member who broke this rule was fined 6d. for each day he detained the book beyond this limit.

These rules are so like those of the present Medical Reading Society, which was established in March, 1807, that I was inclined to think the two were connected, one as the direct successor of the other; and this *may* be the case, for I can find no reference to the "Medical Book Society" after 1807. It was, however, in existence on Feb. 14th, 1807, and at this date the members were the same as at its foundation, except Mr. James Dew; *whereas not one of these* are amongst the eleven original members of the Medical Reading Society. It is, therefore, probable that the two societies were quite distinct. This is almost proved by the fact that Mr. Richard Smith mentions that Richard Lower became a member of this society in 1807, and he must have joined after February 14th, 1807, as his name is not down in the circulating list at that time. It is not likely that he would have joined a few weeks before the dissolution of the society.

### The Medical Reading Society was founded on March 28th, 1807, with similar objects and rules to the Medical Book Society. This is still in full vigour, after the lapse of a hundred and seven years. Some of those now (1914) belonging to the Society remember one or two of the original members. A full and interesting account of this, by Mr. L. M. Griffiths, will be found in the *Bristol Medico-Chirurgical Journal* for September, 1907.

### The Bristol Medico-Chirurgical Society.

This was formed early in the year 1812. Its objects are stated in the following preamble: "That it being the wish of several Medical Gentlemen that a Society should be formed for the promotion of friendly intercourse amongst its members, and for the circulation of Books—resolved: That it be called the Medical and Chirurgical Society, and consist of twelve members only. To meet every third Tuesday at 6 o'clock at each other's houses. Supper each night. Sale of Books in December."

The first meeting was held at Mr. Henry Daniel's house on April 23rd, 1812, and the rules were signed by the following :—

Richard Lowe.
Richard Edgell.
Nathaniel Smith.
James Crang.
Henry Daniel.
Geo. G. Bompas.
Robert Lax (Treasurer).
Robert Dyer.
Samuel Williams.
W. H. Goldwyer.
J. Newman.
Richard Smith.
J. Bedingfield.
James Daly.
Richard Charles Hanson.
William Salmon.

Presumably a ballot was taken, and in the first printed list to be found the above names are down with the exception of Crang, Daly, Hanson, and Salmon. In 1819 the last three appear as members, and Bompas, Dyer and Newman are omitted.

It is uncertain how long this first Medico-Chirurgical Society existed, but there is a letter from William Salmon to Richard Smith, dated "14th Feb., 1825, 23 King's Square," as follows :—

Dear Sir,

The Medical and Chirurgical Society meet at my house to-morrow evening. If you will favour me with your company at supper, I shall be happy in receiving you.

I am,
My dear Sir,
Your obligd. servant,
WILLIAM SALMON.

Another letter, from W. Salmon to R. Smith, dated "Sept. 2nd, 1825," is an invitation to the "Montague" to sup with the members of the *Medical Social Society*. This probably refers to another society, called

### THE MEDICAL AND CHIRURGICAL ASSOCIATION.

which was formed at a quarterly meeting of the Faculty of the Infirmary, held at the Montague Hotel, Kingsdown, on Dec. 8th, 1818. Dr. J. Cowles Prichard and Mr. Richard Smith were deputed "to consider some plan which might promote a friendly intercourse between respectable members of the profession in general," and to report "at the next meeting of the Faculty of the House to be holden on Tuesday, ye 9th of March, 1819." On January 15th, 1819, Messrs. R. Smith, Hetling and Lowe, and Dr. William Gilby, met at Dr. Prichard's house in College Green to consider what means to adopt, and decided to call a meeting of the Faculty at Mr.. Richard Smith's on January 25th, and to invite some other medical men to be present.

At this meeting on the 25th Dr. Carrick (the Senior Physician to the Infirmary) was called to the chair, and it was resolved that "a society be formed under the title of the Medical and Chirurgical Association." The Faculty of the Infirmary were constituted a Committee, with power to add to their number, and the objects of the new society were agreed upon as follows: "To promote a friendly intercourse between the Medical and Surgical Professions in Bristol and its vicinity, and to advance the general interests and respectability of the Profession"—and "in order to promote the interests of the Profession, and in order to obtain a fair and adequate remuneration for attendance it shall be an object of this Association to enter into a general understanding with regard to the sum to be charged for visiting at certain distances."

The Committee met at Dr. Prichard's on March 27th, 1819. They decided "that the most likely method to accomplish the objects desired will be to establish a meeting to be held at the 'Montague' twice a year, in order to dine together: and to ensure a full attendance at each dinner each member is required to deposit in the hands of the Secretary one guinea."

The next meeting of the Committee was at Reeves's Hotel on Saturday, April 3rd, 1819, when Richard Smith was unanimously elected Treasurer and Secretary, and some necessary rules as to election, etc., were passed.

It will be seen that the scope of this Medical and Chirurgical *Association* was different from that of the *Society* of the same name. It was meant to appeal to the profession at large, and had nothing to do with books. It was, in fact, an attempt to organise a social and ethical society for local practitioners.

It will be noticed that the question of fees was to be considered, and it may interest my readers to know that the following resolutions were agreed to:—

1. That the Physicians and Surgeons of the Infirmary and the Senior Members of the Profession generally should consider themselves on a par in regard to charges.
2. The Faculty of the House, together with the Senior Members of the Profession ought to be allowed the cost of a Post Chaise when they go a distance beyond two miles (whether they use their own carriage or not).[1]
3. That a distinction ought to be made between a single visit or two and a regular attendance.
4. All operations are to be paid for independently of the journeys.

[1] The suggested charges for distances were: Clifton, 10s. 6d.; Bedminster, £1 1s.; Redland, £1 1s.; Brislington, Westbury and Ashton Church (each considered about three miles), £1 1s. to £2; Leigh, Henbury and Shirehampton, £2 2s.; Keynsham, Pill, Wraxall, Mangotsfield and Siston(?), £3 3s.; Pensford, £4 4s.; Wrington, £5 5s.

5. Practitioners who furnish and charge for medicines will probably be satisfied with smaller fees for attendance.

6. Less than a guinea ought not to be charged for a visit in the night.

There was an understanding amongst the members that these charges should be adhered to as much as possible, but no one was bound to follow them strictly.

The first dinner was held at the "Montague" on Thursday, May 6th, 1819, at five o'clock. The price was 7s. each, exclusive of wine. The second was held on October the 7th.

The next year (1820) Richard Smith resigned the secretaryship, and Richard Edgell was appointed in his place.

Richard Chafin Edgell, or Edgehill, was born at Frome in 1776. He was indentured to Mr. Joseph Metford as an out-door apprentice for 200 guineas. He became Surgeon to the 2nd Somerset Militia in 1799. He applied three times unsuccessfully for the Surgeoncy of the Infirmary. He had, I believe, no qualification, but enjoyed an extensive practice in medical and midwifery cases, making £1,500 a year. He was an eminent Freemason.

Amongst many letters in answer to the notices of these dinners, I find the following rather curious one :—

DEAR SIR,

It will not be in my power to attend the next meeting of the Medical Association (*sic*).

As there appears to me no particular object in this Society but that of occasionally dining together, for which I have but little time and not much inclination, I will thank you not to insert my name in any list of future subscriptions.

I am,
Yours truly,
JOHN BISHOP ESTLIN.

*April 30th*, 1820.

John Bishop Estlin, M.R.C.S., F.L.S., son of the Rev. John Prior Estlin, LL.D., minister at Lewin's Mead. He was well known and greatly esteemed as the founder of the Eye Dispensary in Frogmore Street, and as a lecturer on Diseases of the Eye and Optics. He also lectured at the Bristol Institution on the " Structure of the Human Body," etc. He applied four times, unsuccessfully, for the post of Surgeon to the Infirmary. His sister married Dr. J. C. Prichard.

This Society lasted some years, but settled down (as intimated in Mr. Estlin's letter) into a social meeting at dinner twice a year, and it does not appear to have done much else.

These purely local societies must not be confounded with the local branch of the Provincial Medical and Chirurgical Association, about which a word or two must be said.

### The Provincial Medical and Chirurgical Association, which was usually, after its beginning, called the Provincial Medical and Surgical Association, was at first confined to the provinces—London was afterwards included—and it ultimately became the British Medical Association.

It was initiated at the Worcester Infirmary on July 19th, 1832, chiefly owing to the exertions of Dr. Charles Hastings, who gave an inaugural address. He and Mr. Sheppard were appointed Secretaries. Dr. Johnstone, of Birmingham, was elected President, and Dr. Carrick, of the Bristol Infirmary, was elected President for the year 1833–4.

A Council was formed, which included four men on the Infirmary staff, viz. Drs. Prichard and Carrick, and Messrs. R. Smith and W. Hetling.

The first anniversary meeting was held at the Infirmary, Bristol, on July 19th, 1833, in the Committee Room. Dr. Carrick, the Senior Physician, was in the chair, and gave an excellent address.

About two hundred members assembled at 11 o'clock. The wards and new out-patient room of the Infirmary were inspected, Dr. Carrick gave his address, and Dr. Hastings read his annual report. Dr. Barlow, of Bath, gave another eloquent address, and various resolutions were passed, and much business transacted. The meeting broke up at half-past 4 o'clock, and the company re-assembled at 6.30 at Ivatt's Hotel, where they sat down to a dinner which did credit to the caterer, the turtle, dessert and wines being very favourably mentioned in the papers. Messrs. Richard Smith and William Hetling acted as Vice-Presidents.

### Formation of a Local Branch.

A proposal to form a local branch of the Association was made at a meeting of doctors in Bristol in June, 1839.

A year after, on June 18th, 1840, a well attended meeting was held at the Medical Library in Orchard Street. Dr. J. C. Prichard was in the chair, and delivered an address, in which he claimed that Bristol was peculiarly fitted "to become the seat of a Medical University," both from its position, the existence of the Infirmary, Hospital and Medical School, and because "the intellectual resources for founding an University are within our reach" (*Felix Farley's Journal*, June 20th, 1840).

When we remember that at this time such men as John Addington Symonds, W. B. Carpenter, William Herapath, and J. C. Prichard were lecturing in Bristol, and were organising and leading medical education in the West of England, we can heartily agree with him.

Dr. Wilson moved at this meeting "that the proposal in June, 1839, for forming the Branch Association be adopted. Mr. Hetling, the Secretary, read the proposed rules, and our friend Richard Smith was elected the first President of the Bristol Branch.

After further business the meeting adjourned until 3 o'clock, when a discussion on puerperal fever was opened by Dr. Symonds, and at 5 o'clock there was a "sumptuous dinner" at the Royal Western Hotel, with Dr. J. C. Prichard in the chair, and Richard Smith as Vice-President. According to the newspaper report "the hours winged their flight during a delightful flow of conversation."

The Bath Branch was founded before this, on Nov. 18th, 1836. The two branches were amalgamated in 1842.[1]

From its foundation until 1863 many Bristol men contributed important papers, especially Symonds, J. C. Prichard, Budd, Wallis, Augustin Prichard, and Davey. Budd made a strong advocacy of photography in medicine at the Torquay meeting in 1860.

The Association met again in Bristol on August 5th, 6th, and 7th, 1863.

In the preliminary notices of the meeting the three hotels mentioned as "among the best" are the "Queen's," the "Bath," and the "White Lion." Dr. Symonds gave the Presidential Address at the Victoria Rooms, and in the evening there was a conversazione, with electrical experiments by Mr. Phillips, of Weston-super-Mare, and a pathological museum was on view, arranged principally by Dr. Samuel Martyn. Supper was served at 11 o'clock. A contributor to the *Bristol Times* said, "I never saw people eat with so great a contempt for the nightmare as did the professional part of the company. . . . Everything you could conceive unwholesome to retire to bed upon, from lobster salad to pork pie, was swept down before the repeated *charges of these dyspepsia defying lancers.* I jogged the elbow of my medical attendant just as he went in for a third helping of raised pie, half a plateful of which would have made me dream that I had the Observatory on, or a plesio-

[1] *Brit. M. J.*, 1882, I. 956.

saurus in, my stomach. 'Pray remember the poor inside,' I said. 'See if you can reach that decanter of sherry for me,' he merely replied, with his mouth half full."[1]

But this meeting of 1863 should be remembered by Bristolians chiefly because of the address in medicine, which was delivered by Dr. William Budd, and was entitled, "Variola Ovina and the laws of contagious Epidemics, illustrated by an experimental type."

In this paper he not only clearly expressed his belief in the recent researches of Pasteur, which had not then gained much ground in England, but he carried the theory of germs as factors in disease to a length never before attempted in this country; and was within a short distance of anticipating Lord Lister's brilliant work.

The address was about an epidemic resembling small-pox in sheep, which ravaged the Wiltshire flocks in the autumn of 1862. It was a contagious eruptive fever, spread, as Budd declared, by flies, starlings, human contact, or in the atmosphere. He reasoned that this poison which killed the animal was produced in the sheep's body, not spontaneously, but from entrance of germs of extreme minuteness and with an "incalculable faculty of growth." He pushed the analogy of this epidemic to the consideration of typhoid and other contagious diseases, and was emphatic in condemning the theory of "spontaneous generation."

[NOTE.—The Provincial Medical and Surgical Association printed *Transactions* from 1833 to 1853. *The Provincial Medical and Surgical Journal* ran from 1840 to 1852. There were thus *two* journals connected with the Association in existence at the same time during a period of twelve years. In 1853 the name of the last was altered to the *Association Medical Journal*, and in 1857 this was again changed to *British Medical Journal*.]

[1] *Bristol M.-Chir. J.*, 1894, xii. 172.

The Association met for the third time in Bristol in 1894. This is so recent that no special notice is required of it here, except to remark that the address in Surgery by Greig Smith, given in Victoria Chapel, will remain in the memories of many who heard it as a masterpiece of eloquence.

### The Bristol Medical Library.

The "Bristol Library Society" was founded on Dec. 15th, 1772. Its chief interest to medical men is that John Ford, Samuel Farr, and Abraham Ludlow, all at that time on the Hon. Medical Staff of the Infirmary, were amongst the most active of the promoters of the scheme.

On Saturday, June 18th, 1831, Dr. Edward Kentish, Mr. T. L. Surrage, and Mr. William Mortimer called upon Richard Smith to discuss the question of forming a medical library. Of these, Dr. Kentish, who founded the "Kentish Baths" near the Cathedral, was the most active organiser, and may be said to have been the founder. He was the first President, and was succeeded, on his death on Dec. 8th, 1832, by Dr. Carrick, who was then Senior Physician to the Infirmary.

Dr. Carrick died on June 14th, 1837, and Dr. J. Cowles Prichard, F.R.S., then Senior Physician to the Infirmary, was elected President on June 26th, at the Annual Meeting. At this time Dr. P. T. Dick was Secretary, Mr. Surrage Treasurer, and Mr. Thomas Green Librarian.

The Library had its head-quarters in Orchard Street in a building formerly used by the French Protestants who came to Bristol after the revocation of the Edict of Nantes. The Society paid the Corporation £2 a year rental.

In 1856 the books were transferred to the Bristol Library, which was in "the west wing of the Bishop's College, on ground now occupied by the Art Gallery."[1]

[1] See Mr. L. M. Griffiths' account of the Medical Library, *Bristol M.-Chir. J.*, 1911, xxix. 338.

After the establishment of the *Bristol Medico-Chirurgical Journal* in 1882, a number of books sent for review and journals sent in exchange, etc., began to accumulate: the urgent need of a Medical Library in some central and convenient situation was felt, and in 1890 these books, together with the remnant of the old Medical Library, were transferred to the newly-formed Literary and Philosophic Club in Berkeley Square.

On July 1st, 1893, the Library was removed to a fine room specially constructed for the purpose in the Medical School Buildings, now forming part of Bristol University.

As the origin of these buildings, at first known as the "New Medical Wing of University College," is becoming "ancient history," it may be as well here to add a few notes on the subject.

The "old" medical wing of University College was a red brick building, described by Dr. Lyon Playfair as "barn-like," part of which still survives. Work was carried on here from 1879 to 1892, with the help of large rooms belonging to the University College. In one of the lecture rooms in this old building a largely-attended meeting of medical students and young practitioners was held on Oct. 28th, 1887. The object was to consider some means of increasing the accommodation at the School. It was decided to represent to the Faculty of Medicine the urgent need for larger and better equipped buildings. A Committee was appointed, of which Mr. Munro Smith was Chairman, Dr. Michell Clarke Treasurer, and Messrs. Mole and Gratte Secretaries.

*On Nov. 4th,* 1887, an interview was granted to this Committee by the Faculty of Medicine of the University College, and a sub-committee of the Faculty were appointed to consider the question.

*On Nov. 15th,* 1887 this sub-committee, together with members of the committee appointed by the students on

Oct. 28th, met at Dr. Markham Skerritt's to consider chiefly the best means of collecting money for the proposed new buildings. It was decided to issue circulars, first to the medical profession in Bristol and neighbourhood, and then to the general public, asking for contributions.

The following day, Nov. 16th, the committee interviewed the Council of University College on the subject.

*On Feb. 16th,* 1888, certain representatives of the conjoint committee of the Faculty and students, viz. Dr. Skerritt, Prof. Lloyd Morgan, Dr. Michell Clarke, and Mr. Munro Smith, met Mr. Albert Fry, Mr. Schacht, and the Secretary of the University College, to discuss "the legal business" connected with the proposed new wing.[1]

*On March 6th* there was a meeting to discuss plans of the building.

In the meanwhile other medical men in Clifton and Bristol had been interested in the subject, and many informal meetings and discussions took place at private houses. Amongst others, Dr. Ed. Long Fox, then Consulting Physician to the Royal Infirmary, took up the matter in a practical manner, and gave a dinner on Saturday, June 2nd, 1888, at the Queen's Hotel, principally to medical men interested in the School.

Dr. Fox, who always made an excellent chairman, gave us a first-rate dinner, and then made a speech in which he laid great stress on the urgent need for better accommodation in the Medical Department of University College. He spoke especially of the importance of having a good museum, and a large room which could be used both as a library and as a place of meeting for the Medico-Chirurgical Society, and the local branch of the Medical Association. Everyone was very enthusiastic, and when a list was sent round no less

[1] These notes are taken chiefly from my diary, and from a sort of minute book which I kept at the time.—G. M. S.

than £1,200 was promised by those present in contribution to the scheme.

*On Dec. 3rd,* 1888, a meeting of the Building Committee was held at the Medical School, and Mr. Hansom, the architect, submitted plans of a four-storied building, with the museum on the second floor, and a library on the ground floor.

*On Dec. 17th,* 1888, Mr. Greig Smith was added to the Building Committee, and objected in his impetuous and energetic way to the plans, which had hitherto been thought good ones; and on Jan. 4th, 1889, a three-story plan, with the Library above and the Museum below, was adopted by the Faculty.

In the meanwhile the medical men of the neighbourhood had been circularised, and by June 7th, 1889, the total subscriptions from the profession alone amounted to £1,709 14s. This money was contributed to the Building Fund, but the real actuating motive was undoubtedly the wish to have a good medical library for the use of the Profession in the neighbourhood, a place for meetings, and a museum; and of these the foremost object was the hope of a permanent room for the Library.

This formed the nucleus of a sum of money which, when added to by grants from the University College, was expended on the new buildings. The individual lecturers had much to say as to the arrangement of their respective departments, but by far the greatest amount of thought was bestowed on the Library.

Amongst other things, stone shields were were placed on the walls, with monograms of well-known Bristol men upon them; and the six chosen were Henry Riley, Henry Clark, William Herapath, John Addington Symonds, Joseph Griffiths Swayne, and William Budd.

Above the entrance to the building is the motto—

"ὁ βίος βραχύς ἡ δὲ τέχνη μακρή."

The new buildings were formally opened on Nov. 16th, 1892.

### Bristol Royal Infirmary Medical Society.

This was founded towards the end of 1850 by students and junior medical men in connection with the Infirmary. Mr. Henry A. Hore, then House Surgeon, was the first President, and Messrs. E. H. Swete and W. Garrard were Hon. Secretaries; Mr. Nathaniel Crisp (elected Assistant House Surgeon in May, 1851), was Vice-President; and Mr. Crosby Leonard, then recently qualified, was the Hon. Treasurer.

From the Annual Report, a modest little pamphlet, with a blue paper cover, we find that at the end of the first session there were thirty-four ordinary members; the Hon. Staff were patrons; and the balance at the end of the year was £3 6s. 9d.

*On January 7th*, 1851, Mr. Harrison, the Senior Surgeon, "asked permission for the use of the Board Room once a fortnight on behalf of a Medical Society recently formed by the students of the institution for promoting a spirit of enquiry into and discussion of, medical topics."

This request was granted by the Committee, on condition that "one of the Physicians or Surgeons, together with the House Surgeon, be present during the whole of the meeting."

Fourteen meetings were held during the session, and were well attended. Interesting subjects were discussed, and two annual prizes were given, one for the best essay on a given topic, and one called "The Discussion Prize," presumably for the best speaker or debater.

Like other societies, it did well so long as it had an energetic secretary. After a few years it languished and ceased, but was revived again in 1869. On March 23rd of that year Mr. Board, the House Surgeon, attended a committee meeting, and asked for a renewal of the privileges granted in 1851. This was agreed to by the Committee, and the Society continued in a moderately thriving condition for some years.

